# Racetrack Ring Resonator-Based on Hybrid Plasmonic Waveguide for Refractive Index Sensing

**DOI:** 10.3390/mi15050610

**Published:** 2024-04-30

**Authors:** Muhammad A. Butt

**Affiliations:** Institute of Microelectronics and Optoelectronics, Warsaw University of Technology, Koszykowa 75, 00-662 Warsaw, Poland; ali.butt@pw.edu.pl

**Keywords:** racetrack ring resonator, integrated optics, silicon-on-insulator, photonic sensor, refractive index sensor

## Abstract

In this study, a comprehensive numerical analysis is conducted on a hybrid plasmonic waveguide (HPWG)-based racetrack ring resonator (RTRR) structure, tailored specifically for refractive index sensing applications. The sensor design optimization yields remarkable results, achieving a sensitivity of 275.7 nm/RIU. Subsequently, the boundaries of sensor performance are pushed even further by integrating a subwavelength grating (SWG) structure into the racetrack configuration, thereby augmenting the light–matter interaction. Of particular note is the pivotal role played by the length of the SWG segment in enhancing device sensitivity. It is observed that a significant sensitivity enhancement can be obtained, with values escalating from 377.1 nm/RIU to 477.7 nm/RIU as the SWG segment length increases from 5 µm to 10 µm, respectively. This investigation underscores the immense potential of HPWG in tandem with SWG for notably enhancing the sensitivity of photonic sensors. These findings not only advance the understanding of these structures but also pave the way for the development of highly efficient sensing devices with unprecedented performance capabilities.

## 1. Introduction

Integrated photonic sensors stand at the forefront of a revolution in medical diagnostics, boasting unmatched sensitivity, miniaturization, and multiplexing capabilities [1]. Leveraging the principles of photonics, these sensors detect subtle changes in biochemical and biophysical parameters critical for disease diagnosis [2]. Through the integration of light sources, waveguides (WGs), and detectors onto a single chip, they facilitate the precise and real-time monitoring of biomarkers in bodily fluids like blood or saliva [2,3]. This groundbreaking technology holds vast potential across various domains, from point-of-care diagnostics to continuous health monitoring and personalized medicine [4,5]. With their remarkable ability to simultaneously detect multiple analytes with exceptional specificity and sensitivity, integrated photonic sensors pave the way for early disease detection, timely intervention, and enhanced patient outcomes [6]. Their compact design and compatibility with existing microfluidic platforms make them ideal for deployment in resource-constrained settings and wearable devices, fundamentally transforming the landscape of medical diagnostics [7]. As ongoing research drives further innovation, integrated photonic sensors are primed to assume a pivotal role in shaping the future of healthcare, offering unparalleled insights into the body’s physiological processes with unprecedented efficiency and precision [8].

Racetrack ring resonators (RTRRs) are emerging as highly promising tools for sensing applications, thanks to their distinctive features [9,10]. These devices comprise a closed-loop WG with two straight and two curved sections, resembling the shape of a racetrack. One of their standout attributes is their remarkable sensitivity to changes in the surrounding environment, such as fluctuations in the refractive index or temperature [10]. This sensitivity arises from the resonant wavelength shift induced by alterations in the effective refractive index within the ring structure. Moreover, RTRRs boast compactness and integration capabilities, making them ideal for deployment in compact sensor arrays tailored for multiplexed sensing [11]. Their ability to confine light within the ring structure for prolonged periods enhances the interaction with analytes, leading to enhanced detection limits. Furthermore, the resonance characteristics of RTRRs can be fine-tuned by adjusting their geometry or utilizing different materials, offering a high degree of versatility in sensor design suitable for a wide range of applications spanning from biochemical sensing to environmental monitoring [12,13].

Hybrid plasmonic waveguides (HPWGs) represent a cutting-edge advancement in nanophotonics, combining the benefits of both plasmonic and dielectric waveguiding structures [14]. These WGs typically consist of a dielectric core surrounded by a thin metal layer, allowing for the efficient confinement and propagation of optical signals at the nanoscale. The high sensitivity of HPWGs originates from their unique ability to tightly confine light within the metal–dielectric interface, enabling strong interactions between light and matter, particularly in the form of surface plasmon polaritons [15]. This enhanced interaction leads to a significant increase in the sensitivity of the WG to changes in its surrounding environment, such as variations in the refractive index or the presence of analytes [16,17]. Moreover, subwavelength grating (SWG) WGs represent a transformative advancement in photonics, holding significant promise for sensing applications [18]. By leveraging the principles of subwavelength nanophotonics, SWG WGs facilitate the precise manipulation and control of light at scales smaller than the light’s wavelength. This unique capability grants SWG WGs exceptional sensitivity to alterations in their surroundings, rendering them highly adept at sensing a wide array of physical, chemical, and biological parameters. Their compact form factor and compatibility with integrated photonic circuits make SWG WGs ideal for on-chip sensing platforms, offering notable advantages in terms of miniaturization, scalability, and seamless integration with other functional components [8,19].

In this paper, an HPWG-based RTRR is proposed and its sensing performance is evaluated in comparison to the SWG-HPWG-based RTRR structure, aiming to enhance its sensing capabilities. The study demonstrates the superiority of the proposed HPWG-based RTRR in sensing performance. Furthermore, a comprehensive review of the existing literature reveals that ring resonator structures implemented on standard WGs [20,21,22] exhibit lower sensitivity compared to the novel approach presented in this work.

## 2. Geometric Optimization of the Sensor’s Design and Simulation Model

**Figure 1:**
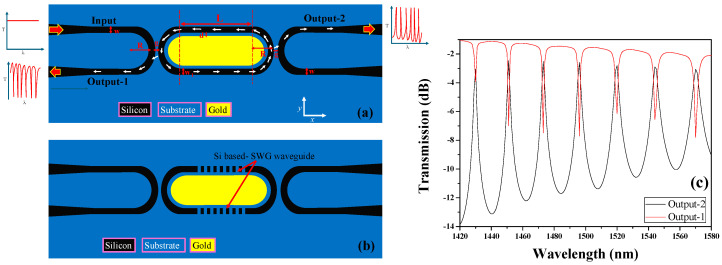
Graphical illustration of (**a**) HPWG-based RTRR, (**b**) SWG-HPWG-based RTRR, (**c**) transmission spectrum from output-1 and output-2. The geometric parameters used for this graph are as follows: R = 2 μm; L = 5 μm; g = 100 nm; d = 80 nm; w = w_1_ = 250 nm.

In this study, two configurations of refractive index sensors are presented: HPWG-based RTRR and SWG-HPWG-based RTRR structures, as illustrated in Figure 1a,b, respectively. Both configurations feature input and output WGs that are side-coupled to the racetrack structure from both sides. The width of the WGs is denoted as ‘w’, while the separation between the racetrack structure and the input/output WGs is denoted as ‘g’. Notably, the ring radius, denoted as ‘R’, remains consistent for both the racetrack and the input/output WGs. The dimensions of the racetrack are denoted as length (L) and width (w_1_). Inside the racetrack, a layer of gold (Au) is deposited, strategically positioned with a small gap “d” separating it from the RTRR. This precise arrangement of materials and geometries enables tailored light–matter interactions within the racetrack structure, enhancing its functionality for enhanced sensing performance. The numerical analysis is conducted based on the geometric parameters and their corresponding ranges, as delineated in Table 1.

Light is initially launched from the input port, where it couples into the racetrack structure under the condition of resonance. Subsequently, the light circulates within the racetrack, undergoing multiple reflections and interactions, before eventually exiting from the output-2 port. This exit occurs in the form of distinctive resonance peaks observed in the transmission spectrum. Conversely, at output-1, resonance dips are obtained, marking points of decreased transmission corresponding to specific resonant wavelengths as shown in Figure 1c. This characteristic behavior of resonance peaks and dips in the transmission spectrum provides valuable insight into the optical properties and performance of the RTRR structure. It is important to highlight that the resonance peaks observed in output-2 exhibit a notably higher extinction ratio (ER), calculated as ER = 10 × log (P^out^/P_in_), compared to the resonance dips observed in output-1. However, this advantage comes with a trade-off: the resonance peaks also entail a wider full width at half maximum (FWHM), which can potentially diminish the overall quality factor (Q-factor) of the device. Hence, in applications such as sensing, the preference leans towards resonance dips characterized by a narrower FWHM for enhanced performance.

### 2.1. Simulation Model

A rigorous simulation endeavor is aimed at dissecting transmittance patterns and field distributions employing the 2D-finite element method (2D-FEM) via COMSOL Multiphysics software (6.1). Leveraging the rich materials library within COMSOL, the refractive indices of pivotal materials such as silicon, air, and gold (Au) are obtained, ensuring reliability to real-world parameters. The simulation protocol entails the precise coupling of TE-polarized light to a 600 nm wide WG, strategically chosen to facilitate seamless coupling and the propagation of single-mode signals within the near-infrared (IR) spectrum. To uphold precision, the device design underwent meticulous segmentation into subdomains, meticulously subdivided into triangular mesh elements. Each element boasts a grid size precisely set at λ/250, guaranteeing an optimal balance between simulation accuracy and computational efficiency. In the pursuit of a realistic electromagnetic wave phenomena analysis, the establishment of an open-bounded domain emerges as a fundamental imperative. To emulate this environment, scattering boundary conditions (SBC) are judiciously implemented along the outer perimeters of the FEM simulation window. Through these methodical approaches and the utilization of computing resources—an 8-core processor (3.8 GHz) paired with 128 GB of RAM—a suite of simulations characterized by their high fidelity and reliability is obtained. These simulations not only provide valuable insights into transmittance characteristics and field distributions but also serve as a cornerstone for the optimization and advancement of photonic device designs.

### 2.2. Optimization Process of HPWG Based on RTRR Structure

**Figure 2:**
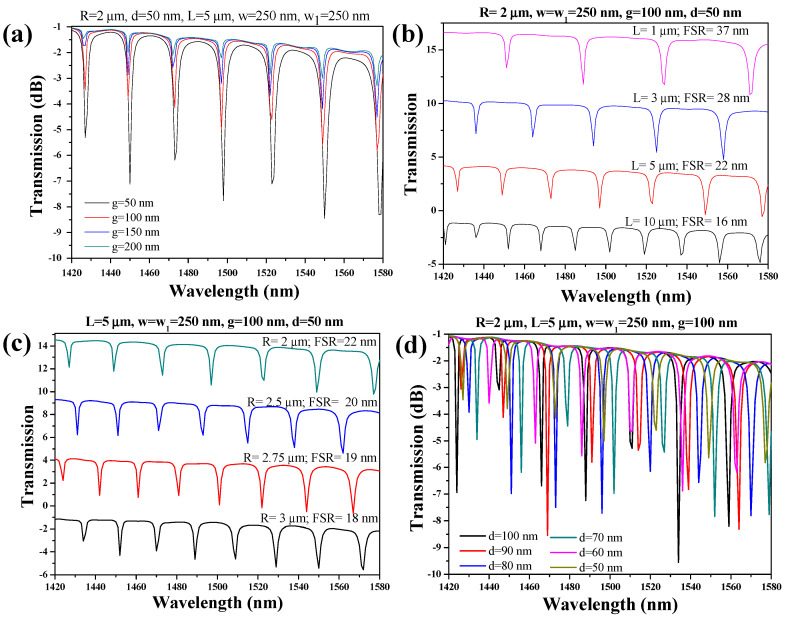
The spectral characteristics of the HPWG-based RTRR structure: (**a**) transmission spectrum versus g, (**b**) FSR versus L, (**c**) FSR versus R, and (**d**) transmission spectrum versus d.

The geometric parametric optimization of the device stands as a pivotal determinant in achieving peak performance for the sensing apparatus. Initially, the focus is directed towards fine-tuning the coupling gap (denoted as ‘g’) between the bus WG and the ring, while maintaining other constant geometric parameters, such as R = 2 µm, d = 50 nm, L = 5 µm, and w = w_1_ = 250 nm. The parameter ‘g’ undergoes variation within the range of 50 nm to 200 nm, allowing for a meticulous examination of the resonance dips within the transmission spectrum, which is depicted across the wavelength span of 1420 nm to 1580 nm. An analysis from Figure 2a reveals a discernible trend: the depth of the resonance dip increases as ‘g’ diminishes from 200 nm to 50 nm. Balancing the imperatives of the fabrication resolution and dimensional flexibility, ‘g’ is ultimately anchored at 100 nm.

The free spectral range (FSR) of a resonator structure is a pivotal parameter that defines its spectral characteristics. It quantifies the frequency separation between neighboring resonance peaks or troughs observed in the resonator’s transmission spectrum. Essentially, it delineates the span of wavelengths across which the resonator displays discernible resonant modes. The FSR is determined by factors such as the disparity in optical path lengths travelled by clockwise and counterclockwise propagating waves within the ring, alongside the effective refractive index of the resonator.

A broader FSR facilitates heightened spectral selectivity, whereas a narrower FSR enables a finer spectral resolution, thus underscoring its critical role in resonator-based photonic device design and optimization. In the RTRR structure, both R and L serve as tunable parameters for adjusting the FSR of the device. Initially, the impact of varying L on the FSR is explored, with the remaining geometric parameters such as R, w, w_1_, g, and d held constant at 2 µm, 250 nm, 250 nm, 100 nm, and 50 nm, respectively. The analysis in Figure 2b reveals that the device exhibits FSR values of 37 nm, 28 nm, 22 nm, and 16 nm for L values of 1 µm, 3 µm, 5 µm, and 10 µm, respectively.

Similarly, the parameter R significantly impacts the FSR of the device as it contributes to the overall path length. To explore this influence, R is systematically adjusted within the range of 2 µm to 3 µm, while maintaining the other geometric parameters—L, w, w_1_, g, and d—at constant values of 5 µm, 250 nm, 250 nm, 100 nm, and 50 nm, respectively. The analysis in Figure 2c reveals a noteworthy variation in the FSR, spanning from 22 nm to 18 nm, as R transitions from 2 µm to 3 µm. This observation underscores the precise control achievable over the device’s FSR through the modulation of parameters such as L and R, thereby enabling tailoring to specific application requirements.

The slot gap (d) in HPWG holds significant importance due to its ability to confine and guide light at the nanoscale. This narrow region between Si and Au allows for a strong interaction between light and matter, enabling efficient light manipulation and control in photonic devices. By harnessing the unique properties of both plasmonic and dielectric materials, the slot gap facilitates the localization of optical fields with unprecedented precision, leading to advancements in sensing, integrated optics, and nanophotonic circuitry.

The parameter ‘d’ is systematically varied within the range of 50 nm to 100 nm, maintaining constant values for the remaining geometric parameters, including R = 2 µm, L = 5 µm, g = 100 nm, and w = w_1_ = 250 nm. In Figure 2d, the transmission spectrum of the HPWG-based RTRR is depicted, revealing that the resonance dip experiences minimal variation in ER as ‘d’ is adjusted. Considering fabrication constraints and minor inaccuracies, a value of 80 nm is selected for ‘d’, culminating in the attainment of an ER of −6.3 dB ensuring both practical fabrication feasibility and desired sensing performance.

## 3. Results and Discussion of the Performance of Both Sensor Architectures

In this section, the sensing performance of the proposed devices is comprehensively discussed to highlight their significance and potential utilization as a label-free biosensor.

### 3.1. Significance of Refractive Index Sensing

Refractive index sensors serve as vital tools in biosensing, thanks to their capacity to discern subtle changes in the refractive index of biological samples, thus holding significant implications for a variety of biomedical applications [23,24,25]. The importance of refractive index sensors in biosensing lies in their ability to offer label-free, real-time detection of biomolecular interactions, eliminating the necessity for exogenous labels or reagents [26]. This label-free approach not only streamlines experimental procedures but also minimizes potential artifacts, making refractive index sensors especially valuable for investigating intricate biological systems [27]. Within biosensing, these sensors exhibit the capability to detect biomolecules such as proteins, nucleic acids, and cells with exceptional sensitivity and specificity, providing valuable insights into biological processes, disease mechanisms, and interactions with drugs [13,28]. Furthermore, refractive index sensors enable the development of compact, portable devices ideal for point-of-care diagnostics, personalized medicine, and biomedical research endeavors [29].

### 3.2. Parameters to Determine the Sensor’s Performance

The sensitivity of a refractive index sensor denotes its capacity to discern minute variations in the refractive index of a substance, often triggered by external stimuli or changes within the medium under observation, and can be calculated as [30]:

(1) $$Sensitivity\left(S\right)=\frac{∆\lambda }{∆n};$$

where ∆λ and ∆n are the change in resonance wavelength and change in ambient refractive index, respectively. This attribute holds paramount importance as it directly influences the accuracy and dependability of the sensor across a spectrum of applications including biochemical analyses, environmental monitoring, and industrial quality assurance. A refractive index sensor boasting high sensitivity can perceive even the most subtle shifts in the refractive index, facilitating the thorough measurement and analysis of the target material [31]. Another important factor known as the Q-factor plays a pivotal role in analyzing resonant systems, particularly in the context of RRs [10,32]. This parameter represents the relationship between the energy stored within the resonator and the energy dissipated per cycle. It can be calculated as follows [19]:

(2) $$Q-factor=\frac{\lambda_{res}}{FWHM};$$

where λ_res_ denotes the resonance wavelength and FWHM represents the full width at half maximum. A higher Q-factor signifies diminished energy dissipation and a more confined spectrum of resonant frequencies, thereby enhancing the accuracy and efficiency of filtering or sensing tasks [33]. Such efforts are crucial for developing advanced RR-based technologies across diverse domains like telecommunications, photonics, and sensing applications.

### 3.3. Transmission in the Presence of Varying Ambient Medium Refractive Index

**Figure 3:**
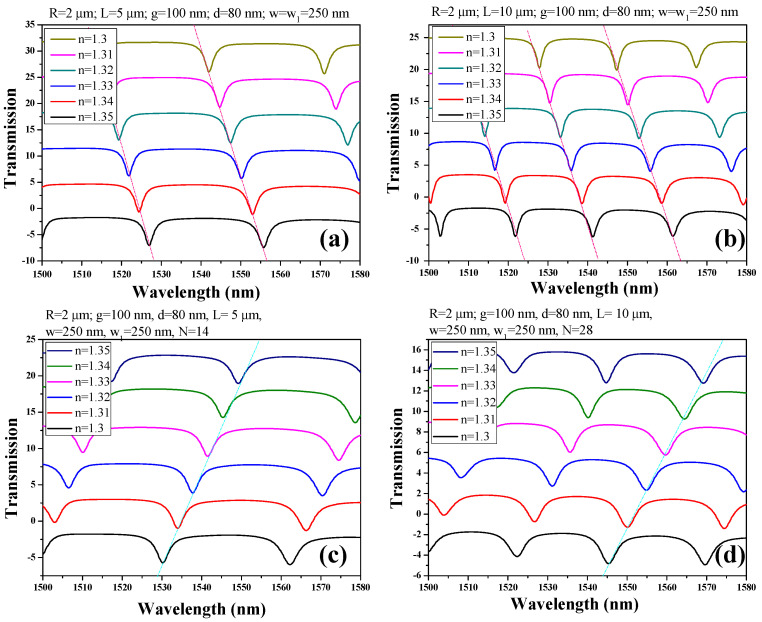
Transmission spectrum of (**a**) HPWG-based RTRR of L = 5 μm, (**b**) HPWG-based RTRR of L = 10 μm, (**c**) SWG-HPWG-based RTRR of L = 5 μm, and (**d**) SWG-HPWG-based RTRR of L = 10 μm.

For SWG-HPWG-based RTRR, the optimal grating period is determined from [19], utilizing a period (Λ) of 330 nm with a fill factor (FF) of 0.76. The number of periods (N) is set as 14 and 28 for L equal to 5 μm and 10 μm, respectively. It is important to note that while these values provide a starting point, they are not rigid constraints: L and N can be adjusted according to the desired footprint of the device. The transmission spectra of both HPWG-based RTRR and SWG-HPWG-based RTRR for L = 5 µm and 10 µm are showcased in Figure 3a–d. The resonance dips exhibit a notable redshift as the refractive index within the sensing area ranges from 1.3 to 1.35. The magnitude of this resonance wavelength shift is intricately tied to the sensitivity of the device, a parameter quantifiable through the utilization of the Equation (1). This observation underscores the crucial role of sensitivity in discerning subtle changes in the refractive index, and highlights its significance in optimizing the performance of the device for precise sensing applications.

### 3.4. Field Distribution in the Device at On-Resonance and Off-Resonance States

**Figure 4:**
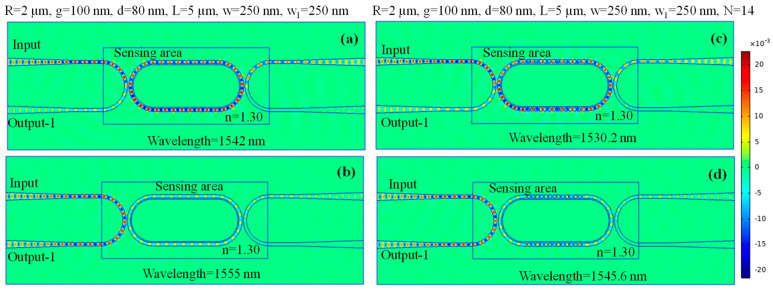
Normalized H-field distribution in the HPWG-based RTRR structure at (**a**) on-resonance state, and (**b**) off-resonance state. Normalized H-field distribution in the SWG-HPWG-based RTRR structure at (**c**) on-resonance state, and (**d**) off-resonance state.

The normalized H-field distribution is depicted for the HPWG-based RTRR, illustrating both the on-resonance and off-resonance states in Figure 4a,b, respectively. Furthermore, the H-field distribution is presented for the SWG-HPWG-based RTRR, showcasing the on-resonance and off-resonance states in Figure 4c,d, respectively. It is observable that under resonance conditions, light exits through output-2, while during the off-resonance state, light exits through output-1.

### 3.5. Comparison of Sensor Device Architectures in Terms of Sensitivity

The geometric parameters, including R, w, w_1_, g, d, and L, are methodically maintained at identical values across both sensor configurations, ensuring a fair comparison of sensitivity. Among these parameters, the length (L) of the racetrack structure emerges as pivotal, profoundly influencing the light–matter interaction and thereby amplifying the device’s sensitivity. To evaluate this impact comprehensively, L is varied at 5 µm and 10 µm, allowing for a thorough examination of sensitivity across different structural lengths.

Hence, four distinct cases are explored: (I) HPWG-based RTRR with L = 5 µm, (II) HPWG-based RTRR with L = 10 µm, (III) SWG-HPWG-based RTRR with L = 5 µm, and (IV) SWG-HPWG-based RTRR with L = 10 µm. It is essential to highlight that for the SWG-HPWG-based RTRR design, the number of periods (N) of the SWG at 14 and 28 for L = 5 µm and 10 µm are maintained, respectively. This careful control ensures consistency in the SWG configuration across different lengths, enabling a comprehensive comparison of the sensor performance under varied structural conditions.

**Figure 5:**
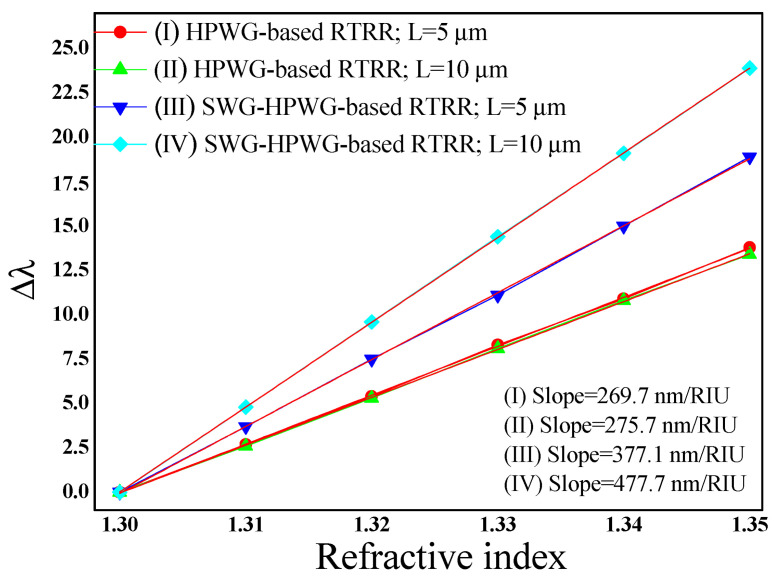
Change in resonance wavelength versus refractive index.

Figure 5 illustrates the shift in resonance wavelength concerning the refractive index across the four aforementioned cases. Notably, the sensitivity of the SWG-HPWG-based RTRR escalates from 377.1 nm/RIU to 477.7 nm/RIU with an increase in L from 5 µm to 10 µm. In contrast, the influence of L on the sensitivity of the HPWG-based RTRR structure appears minimal, with sensitivity hovering around 269.7 nm/RIU and 275.7 nm/RIU for the L values of 5 µm and 10 µm, respectively. This observation underscores the differential impact of structural variations on sensitivity between the two configurations, highlighting the unique advantages conferred by the SWG-HPWG-based RTRR design in enhancing sensitivity with an increased path length. Table 2 provides the main performance indicators of the proposed devices.

The sensitivity of the device achieved in this work has been compared with that reported in previously published papers to demonstrate the significance of this work. A high-sensitivity complex refractive index sensing device is proposed and experimentally demonstrated utilizing an SWG microring resonator exhibiting a sharp Fano resonance at a wavelength of 1550 nm. The microring is composed of trapezoidal silicon pillars with a subwavelength period designed to enhance the overlap between light and analytes, resulting in a high-quality factor. Additionally, the sensor’s capability to detect glucose solution concentrations is experimentally demonstrated, achieving a high experimental sensitivity of 363 nm/refractive index unit (RIU) [34]. A slot waveguide-based ring resonator in silicon on insulator (SOI) is presented and fabricated with optical lithography. Experiments have shown that it exhibits 298 nm/RIU sensitivity for changes in the refractive index of the top cladding [35]. Wang et al. proposed a novel silicon photonic biosensor using phase-shifted Bragg gratings in a slot waveguide. The optical field was concentrated inside the slot region, resulting in efficient light–matter interaction. The Bragg gratings were formed with sidewall corrugations on the outside of the waveguide, and a phase shift was introduced to create a sharp resonant peak within the stop band. A high sensitivity of 340 nm/RIU was experimentally demonstrated and measured in salt solutions [36]. Fard et al. investigated, both by simulations and experiments, ultra-thin TE resonator sensors within the constraint of available thicknesses in standard MPW foundries and services. Sensitivities exceeding 100 nm/RIU were obtained with the ultra-thin TE resonator sensors. The increased stability of these ultra-thin resonators, compared to the traditional 220 nm thick resonators, has been demonstrated by experiments and simulations in the presence of temperature variations [37].

### 3.6. Anticipated Optical Characterization Setup

**Figure 6:**
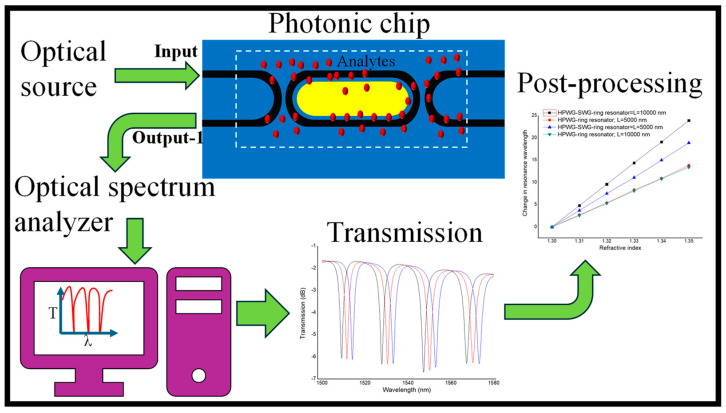
The graphic illustration of the anticipated sensing mechanism.

The outlined experimental procedure is depicted in Figure 6. Initially, TE-polarized light emitted from the tunable laser source is directed to couple with the input WG via a tapered optical fiber. To maintain the desired polarization, a 3-paddle polarization controller is strategically inserted between the laser source and the output end of the fiber (not shown in the figure). Subsequently, the resultant output light is collected from output-1 and routed to the optical spectrum analyzer (OSA). Seamless data acquisition and an analysis are facilitated by connecting a computer to the OSA, thus enabling the automation of measurement protocols and remote instrument control. This integration empowers users to program specific measurement sequences, schedule tasks, and manage the instrument remotely, thereby streamlining operations and enhancing overall efficiency. Following data acquisition, post-processing procedures are employed to extract and analyze the spectral characteristics of the device, enabling comprehensive evaluations and further insights into its performance.

## 4. Concluding Remarks

In conclusion, the HPWG-based RTRR presents a promising platform for refractive index sensing applications. Through leveraging the unique properties of plasmonic materials and WGs, this device offers significant advantages such as high sensitivity, a compact footprint, and compatibility with integrated photonic circuits. By confining light within the resonator structure, it enables the precise detection of minute changes in the refractive index, making it suitable for various sensing applications including environmental monitoring, biomedical diagnostics, and chemical sensing. The RTRR geometry provides enhanced interaction between the guided light and the surrounding medium, leading to an improved sensitivity of 275.7 nm/RIU compared to traditional WG-based sensors. Moreover, the sensitivity of the device can be significantly augmented by integrating SWG WGs into the RTRR structure. This incorporation results in a remarkable augmentation, advancing the sensitivity up to 477.7 nm/RIU. These findings not only underscore the potential of HPWG and SWG WG structures, but they also offer crucial insights for designing next-generation photonic sensors with unparalleled sensitivity. Thus, the integration of SWG WGs into the HPWG-based RTRR architecture represents a significant advancement in the quest for high-performance photonic sensors, poised to revolutionize fields ranging from healthcare to environmental monitoring and beyond.

## Figures and Tables

**Table 1:** Geometric parameters used in this study.

Parameters	Expression	Value
R	Radius of both ring and bus WGs	2 µm
L	Pathlength of the RTRR	1 µm, 3 µm, 5 µm, 10 µm
w	Width of bus WG	250 nm
w_1_	Width of a ring structure	250 nm
g	Gap between bus WG and ring	50, 100, 150, 200 nm
d	Nanoslot	50, 60, 70, 80, 90, 100 nm
Λ = a + b	Period of SWG	330 nm
FF	Fill factor	b/Λ = 0.76

**Table 2:** Performance of the HPWG-based RTRR and SWG-HPWG-based RTRR structures.

	HPWG-Based RTRR		SWG-HPWG-Based RTRR	
L (µm)	5	10	5	10
Sensitivity (nm/RIU)	269.7	275.7	377.1	477.7
Resonance wavelength (nm)	1497	1492	1564	1559.3
FWHM (nm)	1.61	1.42	5	4.5
Q-factor	930	1050	312.8	346.5

## Data Availability

The original contributions presented in the study are included in the article, further inquiries can be directed to the corresponding author.

## Abbreviations

Racetrack ring resonator = RTRR; hybrid plasmonic waveguide = HPWG; subwavelength grating = SWG.
